# Supplementary material for: Autosomal recessive hyposegmentation of granulocytes in Australian Shepherd Dogs indicates a role for LMBR1L in myeloid leukocytes
Source: PLoS Genet. 2023 Jun 22;19(6):e1010805. doi: 10.1371/journal.pgen.1010805 (PMC10321630; doi:10.1371/journal.pgen.1010805)
Supplement: S1 Fig — (PDF) [file pgen.1010805.s001.pdf]

S1 Figure. Re-analysis of the PHA incidence data in F1 progeny from parents with a known granulocyte phenotype originally reported in Table 2 of Latimer *et al.* [1]. We repeated the analysis of Latimer *et al.* and compared the observed incidences with the expected incidences under a semi-dominant mode of inheritance assuming embryonic lethality of the homozygotes. We additionally compared the observed data to the expected incidences for a fully dominant and a fully recessive mode of inheritance. Expected incidences that are compatible with the observed data are indicated in green. Expected incidences that are not compatible with the data are indicated in red.

				Model 1: autosomal semi-dominant inheritance with homozygous lethality[a]	Model 2: autosomal dominant inheritance[a]	Model 3: autosomal recessive inheritance[a]
Phenotype of parents	Observed PHA phenotype in offspring	Observed normal phenotype in offspring	Observed incidence of PHA	Expected incidence of PHA phenotype	Expected incidence of PHA phenotype	Expected incidence of PHA phenotype
PHA x PHA	12	0	100%	**67%**	**75% … 100%**	**100%**
PHA x Normal	26	61	30.0%	**50%**	**50% … 100%**	**0 … 50%**
Normal x Normal	6	149	3.9%	**0**	**0**	**0 … 25%**

[a]Genotype-phenotype correlations for the 3 different models

Genotype	Phenotype (model 1, semi-dominant)	Phenotype (model 2, autosomal dominant)	Phenotype (model 3, autosomal recessive)
wildtype/wildtype	normal	normal	normal
wildtype/mutant	PHA (live dog, hyposegmented neutrophils)	PHA (live dog, hyposegmented neutrophils)	normal
mutant/mutant	embryonic lethal	PHA (live dog, hyposegmented neutrophils)	PHA (live dog, hyposegmented neutrophils)

Reference

1. Latimer KS, Campagnoli RP, Danilenko DM. Pelger-Huët Anomaly in Australian Shepherds: 87 Cases (1991 - 1997). *Comparative Haematology International* 2000;10, 9–13.
