# Supplementary material for: Autosomal recessive hyposegmentation of granulocytes in Australian Shepherd Dogs indicates a role for LMBR1L in myeloid leukocytes
Source: PLoS Genet. 2023 Jun 22;19(6):e1010805. doi: 10.1371/journal.pgen.1010805 (PMC10321630; doi:10.1371/journal.pgen.1010805)
Supplement: Attachment — Submitted filename: 2023May12_Cover_letter_R1.pdf [file pgen.1010805.s013.pdf]

UNIVERSITÄT BERN

Institut für Genetik, Bremgartenstr. 109a, CH-3001 Bern

Institute of Genetics
Vetsuisse Faculty

Director

Editor
PLoS Genetics

Bern, 12-May-2023

Revised manuscript for publication in *PLoS Genetics*, PGENETICS-D-21-00621

Dear Dr. Barsh, dear Dr. Copenhaver,

Thank you very much for the positive evaluation of our manuscript entitled **„Autosomal recessive hyposegmentation of granulocytes in Australian Shepherd Dogs indicates a role for *LMBR1L* in myeloid leukocytes"**. Please find enclosed our revised manuscript. With respect to the comments we made the following changes:

Reviewer 1:

(1)
I am intrigued by the high frequency of the variant since it appears to have occurred after breed divergence and causes an undesirable phenotype. According to GeneCards, this gene is regularly reported in GWAS for body size/weight. Maybe the allele is under selection for size in ASD, or subpopulations of ASD?
Response: We are not able to make any statements about other phenotypic consequence of the *LMBR1L* mutant genotype than the alteration of granulocyte morphology. It is not really clear whether the *LMBR1L* variant truly causes an "undesirable phenotype" in dogs. We agree with the reviewer that a thorough investigation whether the *LMBR1L* mutant genotype has any consequences for health and/or morphology is indicated, but this is beyond the focus of our study and should be addressed in dedicated future investigations.
The *LMBR1L* gene is located very close to *KMT2D* (encoding a histone methylase), *WNT1* and *WNT10A*. In a recent publication reporting "a saturated map" of human height loci, no SNV within *LMBR1L* was significantly associated with height. However, rs2241726, a silent coding SNV within *KMT2D* was significantly associated with human height (Yengo *et al.* (2022) *Nature* 610:704-712). We speculate that the association between *LMBR1L* and size reported in GeneCards may have been spurious and due to the close physical proximity of *LMBR1L* and *KMT2D*.

Prof. Dr. Tosso Leeb
Institut für Genetik
Bremgartenstr. 109a, Postfach
CH-3001 Bern

Tel +41 31 631 23 26
Fax +41 31 631 26 40
Tosso.Leeb@vetsuisse.unibe.ch
www.genetics.unibe.ch

We do not have any data suggesting that the high frequency of the mutant *LMBR1L* allele in ASDs is due to selection for size or any other morphological phenotype. The mutant *LMBR1L* allele has been segregating in this breed for decades. Given the small effective population size of pure dog breeds, major changes in allele frequency are common and often due to genetic drift.

(2)
The author summary should be non-technical and appropriate for a wide audience. Parts of your author summary are more technical than the abstract (e.g., the opening sentences).
Response: We slightly modified the author summary and the abstract and hope that the style of these sections is now appropriate.

(3)
I appreciate that you are telling the story as it unfolded, but I found it a bit confusing/misleading that the summaries start by talking about PHA and Greenberg but then the story ends up not really being about either one. I also don’t like the emphasis in the summaries on how these results “refute previous hypothesis on the mode of inheritance.” I think it is fine to discuss, but the wording in the abstract and summary feels strong and distracts from the importance of the work. Additionally, the resorbed and stillborn puppies in the litter give me pause and make me wonder if, while it looks a lot like a simple recessive, maybe there IS more to it? Are there modifiers? Is it semilethal? The excessive number of stillborn puppies is what prompted the study so naturally it feels disappointing that the conclusion of the study is that the stillbirths are unrelated to the findings.
Response: Thank you for this comment. We had originally thought of submitting a much shorter and more streamlined manuscript omitting the investigation of the litter with the stillborn puppies that indeed do not contribute to our understanding of the hematological phenotype and *LMBR1L* function. After some reflection, we came to the conclusion that the full description of the true historic order of events during this research project might be of interest to some readers. It illustrates that starting hypotheses in hypothesis-driven research may turn out to be false and that hypothesis-driven and hypotheses-free research methods can nicely complement each other.
In the manuscript, we also tried to balance the (basic) scientific advance regarding our knowledge on *LMBR1L* function with the use-inspired applied aspects. The manuscript will not only be read by (basic) scientists, but also by veterinarians and dog owners/breeders. For this audience, it is important that they understand that the literature knowledge on "Pelger-Huët anomaly" in Australian Shepherd Dogs has been incorrect for the last ~30 years.
We hope that you agree with our reasoning and allow us to keep the organization of the manuscript. We removed the strong wording ("refute previous hypotheses") from the abstract and author summary. It is indeed sufficient, if these parts are limited to the discussion.

(4)
In the diagnosis of HG, is it all or none? How many abnormal cells need to be present to classify as affected?
Response: The HG phenotype is NOT an all or none phenotype. Even HG affected dogs have a small proportion of granulocytes with inconspicuous nuclear morphology. We classified dogs as HG affected, when the majority of granulocytes showed hyposegmented nuclei. We added more details to the methods sections to make this clear.

(5)
Were the parents of the litter closely related?
Response: The parents of the litter with the stillborn puppies did not share any common ancestors within three generations and were not particularly closely related. Nonetheless, they were purebred Australian Shepherd Dogs and with purebred dogs, it is inevitable that they must have some common ancestors further in the past. We added this information to the Methods section at the end of the manuscript.

(6)
The bands in Figure 1A are challenging to see.
Response: Figure 1A does not contain any bands. Did the reviewer perhaps refer to figure 4A, which indeed has some faint bands?
We provide a revised version for Figure 4, which includes a contrast-enhanced version of the image with the bands. Please note that we altered brightness and contrast of the (entire) original image in Photoshop to increase the visibility of the bands. We considered this acceptable as the "original image" was already a computer-generated representation resembling a conventional agarose gel that was produced by the instrument software on the Fragment Analyzer capillary gel electrophoresis instrument. If you consider this an inappropriate image manipulation, please use the original version of figure 4.

All changes in the manuscript are in indicated in Microsoft Word tracking mode. If you have any further questions regarding our manuscript, please do not hesitate to contact me at any time. We are looking forward to hearing your decision whether the revised manuscript can now be published in *PLoS Genetics.*

Yours sincerely,

Tosso Leeb

Tosso Leeb
